# Sex-specific associations of comorbidome and pulmorbidome with mortality in chronic obstructive pulmonary disease: results from COSYCONET

**DOI:** 10.1038/s41598-022-12828-8

**Published:** 2022-05-24

**Authors:** Franziska C. Trudzinski, Rudolf A. Jörres, Peter Alter, Julia Walter, Henrik Watz, Andrea Koch, Matthias John, Marek Lommatzsch, Claus F. Vogelmeier, Hans-Ulrich Kauczor, Tobias Welte, Jürgen Behr, Amanda Tufman, Robert Bals, Felix J. F. Herth, Kathrin Kahnert, The COSYCONET Study Group, Stefan Andreas, Robert Bals, Jürgen Behr, Kathrin Kahnert, Burkhard Bewig, Roland Buhl, Ralf Ewert, Beate Stubbe, Joachim H. Ficker, Manfred Gogol, Christian Grohé, Rainer Hauck, Matthias Held, Berthold Jany, Markus Henke, Felix Herth, Gerd Höffken, Hugo A. Katus, Anne-Marie Kirsten, Henrik Watz, Rembert Koczulla, Klaus Kenn, Juliane Kronsbein, Cornelia Kropf-Sanchen, Christoph Lange, Peter Zabel, Michael Pfeifer, Winfried J. Randerath, Werner Seeger, Michael Studnicka, Christian Taube, Helmut Teschler, Hartmut Timmermann, J. Christian Virchow, Claus Vogelmeier, Ulrich Wagner, Tobias Welte, Hubert Wirtz

**Affiliations:** 1 Department of Pneumology and Critical Care Medicine, Thoraxklinik University of Heidelberg, Translational Lung Research Center Heidelberg (TLRC-H), German Center for Lung Research (DZL), Röntgenstrasse 1, 69126 Heidelberg, Germany; 2 Institute and Outpatient Clinic for Occupational, Social and Environmental Medicine, Ludwig Maximilians University (LMU), Comprehensive Pneumology Center Munich (CPC-M), German Center for Lung Research (DZL), Munich, Germany; 3 Department of Medicine, Pulmonary and Critical Care Medicine, Philipps University of Marburg (UMR), German Center for Lung Research (DZL), Marburg, Germany; 4 Department of Medicine V, University Hospital, Comprehensive Pneumology Center Munich (CPC-M), Member of the German Center for Lung Research (DZL), LMU Munich, Ziemssenstraße 1, 80336 Munich, Germany; 5 Pulmonary Research Institute at LungenClinic Grosshansdorf, Airway Research Center North (ARCN), German Center for Lung Research (DZL), Woehrendamm 80, 22927 Grosshansdorf, Germany; 6 Pyhrn-Eisenwurzen-Klinikum Steyr, Klinik Für Pneumologie, Lehrkrankenhaus Der Uniklinik Linz, Sierninger Str. 170, 4400 Steyr, Austria; 7 Praxis Für Pneumologie Am Asklepios Klinikum Uckermark, Schwedt, Germany; 8 Abteilung Für Pneumologie, Interdisziplinäre Internistische Intensivstation, Medizinische Klinik I, Zentrum Für Innere Medizin, Universitätsmedizin Rostock, Rostock, Germany; 9 Department of Diagnostic and Interventional Radiology, University Hospital of Heidelberg, Translational Lung Research Center Heidelberg (TLRC-H), German Center for Lung Research (DZL), Heidelberg, Germany; 10 Department of Pneumology, Hannover Medical School, Carl-Neuberg-Str. 1, 30625 Hannover, Germany; 11 Department of Internal Medicine V - Pulmonology, Allergology, Critical Care Care Medicine, Saarland University Hospital, Homburg, Germany; 12 Lungenfachklinik, Immenhausen, Germany; 13 Universitätsklinikum Des Saarlandes, Homburg, Germany; 14 Klinikum Der Ludwig-Maximilians-Universität München, Munich, Germany; 15 Universitätsklinikum Schleswig Holstein, Kiel, Germany; 16 Universitätsmedizin Der Johannes-Gutenberg-Universität Mainz, Mainz, Germany; 17 Universitätsmedizin Greifswald, Greifswald, Germany; 18 Klinikum Nürnberg, Paracelsus Medizinische Privatuniversität Nürnberg, Nuremberg, Germany; 19 Institut Für Gerontologie, Universität Heidelberg, Heidelberg, Germany; 20 Ev. Lungenklinik Berlin, Berlin, Germany; 21 Kliniken Südostbayern AG, Kreisklinik Bad Reichenhall, Bad Reichenhall, Germany; 22 Klinikum Würzburg Mitte gGmbH, Standort Missioklinik, Würzburg, Germany; 23 Asklepios Fachkliniken München-Gauting, Gauting, Germany; 24 Thoraxklinik Heidelberg gGmbH, Heidelberg, Germany; 25 Fachkrankenhaus Coswig GmbH, Coswig, Germany; 26 Universitätsklinikum Heidelberg, Heidelberg, Germany; 27 Pneumologisches Forschungsinstitut an Der Lungenclinic Grosshansdorf GmbH, Großhansdorf, Germany; 28 Schön Klinik Berchtesgadener Land, Schönau Am Königsee, Germany; 29 Berufsgenossenschaftliches Universitätsklinikum Bergmannsheil, Bochum, Germany; 30 Universitätsklinikum Ulm, Ulm, Germany; 31 Forschungszentrum Borstel, Sülfeld, Germany; 32 Klinik Donaustauf, Donaustauf, Germany; 33 Wissenschaftliches Institut Bethanien E. V., Solingen, Germany; 34 Justus-Liebig-Universität Gießen, Giessen, Germany; 35 Uniklinikum Salzburg, Salzburg, Austria; 36 Ruhrlandklinik gGmbH Essen, Essen, Germany; 37 Hamburger Institut Für Therapieforschung GmbH, Hamburg, Germany; 38 Universitätsklinikum Rostock, Rostock, Germany; 39 Universitätsklinikum Gießen und Marburg GmbH, Marburg, Germany; 40 Klinik Löwenstein gGmbH, Löwenstein, Germany; 41 Medizinische Hochschule Hannover, Hannover, Germany; 42 Universitätsklinikum Leipzig, Leipzig, Germany

**Keywords:** Respiratory tract diseases, Medical research, Prognosis

## Abstract

In patients with COPD, it has not been comprehensively assessed whether the predictive value of comorbidities for mortality differs between men and women. We therefore aimed to examine sex differences of COPD comorbidities in regard with prognosis by classifying comorbidities into a comorbidome related to extrapulmonary disorders and a pulmorbidome, referring to pulmonary disorders. The study population comprised 1044 women and 1531 men with the diagnosis of COPD from COSYCONET, among them 2175 of GOLD grades 1–4 and 400 at risk. Associations of comorbidities with mortality were studied using Cox regression analysis for men and women separately. During the follow-up (median 3.7 years) 59 women and 159 men died. In men, obesity, hypertension, coronary artery disease, liver cirrhosis, osteoporosis, kidney disease, anaemia and increased heart rate (HR) predict mortality, in women heart failure, hyperuricemia, mental disorders, kidney disease and increased HR (*p* < 0.05 each). Regarding the pulmorbidome, significant predictors in men were impairment in diffusion capacity and hyperinflation, in women asthma and hyperinflation. Similar results were obtained when repeating the analyses in GOLD 1–4 patients only. Gender differences should be considered in COPD risk assessment for a tailored approach towards the treatment of COPD.

Clinical Trial Registration: ClinicalTrials.gov NCT01245933.

## Introduction

Among the chronic diseases with high prevalence, chronic obstructive pulmonary disease (COPD) is of major importance^1^. Regarding the pattern of symptoms^2^ and prevalence of comorbidities^3,4^, differences between men and women have been described^4–7^. The same is true for the relationship between parameters, for example regarding the association between symptoms and cardiac disease^8^. Several studies also reported different lung function patterns in men and women^7^ and a higher exacerbation frequency in women^5^, affecting long-term survival^9^.

Comorbidities, defined as clinical diagnosis and/or via biochemical markers, are important predictors of the course of COPD, especially mortality risk. Their role was impressively shown in a comprehensive analysis by Divo and colleagues^10^ that included a wide variety of disorders summarized as „comorbidome “. This analysis relied on a large population (n = 1664) which was, however, dominated by men (89% of participants); consequently, no separate analysis for men and women was performed. Many COPD patients die from extra-pulmonary causes, in particular cardiac disorders^11^. This could be relevant as women show a lower prevalence of cardiac disorders than men^8,12^.

Accordingly, women participating in the “Toward a Revolution in COPD Health study” had lower mortality rates^7^. If, however, the longer life expectancy of women is taken into account, the COPD-attributed loss is higher for women^13^. In the United States, the number of women dying from COPD already exceeded that of men in 2000^14^. This raises the question which other sex-specific differences in common comorbidities are relevant in women versus men. For a comprehensive analysis, it could be of advantage to categorize comorbidities into those referring to the respiratory system and those of extra-pulmonary origin. The latter can be termed “comorbidome” as proposed earlier^10^, while the former might be termed “pulmorbidome”.

Based on these considerations, we investigated differences in the predictive value of comorbidities between men and women using data from an established, large COPD cohort^15^. This cohort comprised 41% women, thereby allowing for a comparative analysis for men and women with similar statistical power. The cohort provided a detailed assessment of comorbidities, clinical and functional state, and mortality over a median follow-up period of up to 3.7 years.

## Methods

### Study population

Data from the prospective COPD cohort COSYCONET ("COPD and SYstemic consequences-COmorbidities NETwork") were analysed. In this cohort, 2741 patients had been enrolled from 2010–2013, including patients from GOLD grades 1–4^15^, but also patients with the diagnosis of COPD who did not fit into GOLD 1–4 grades, particularly of the former Global Initiative for Chronic Obstructive Lung Disease (GOLD) grade 0 (COPD at risk)^16^. Inclusion and exclusion criteria, study protocol and assessments have been published elsewhere^15^; for the present analyses, it is important, that patients with a previous diagnosis of cancer including lung cancer were excluded. For the current analysis, we required complete data on spirometry used for COPD grading, comorbidities, and the laboratory parameters creatinine, hemoglobin and uric acid used to define comorbidities.

### Assessments

According to the COSYCONET study protocol^15^, (spirometry, bodyplethysmography diffusing capacity for carbon monoxide (CO)) were performed following established recommendations. Reference values from the Global Lung Function Initiative (GLI) or European Coal and Steel Community (ECSC) were used^17–19^. Besides forced expiratory volume in 1 s (FEV_1_), forced vital capacity (FVC) and their ratio FEV_1_/FVC, we used functional residual capacity (FRC) and total lung capacity (TLC), as well as their ratio FRC/TLC. For diffusion capacity, we used the transfer factor DLCO. The classification into GOLD groups^20^ was based on the modified Medical Research Council (mMRC) scale^21^. Comorbidities were either recorded in structured interviews based on physician-based diagnoses or defined based on disease-specific biomarkers (see below).

### Definition of the pulmorbidome

The pulmorbidome comprised asthma, chronic bronchitis, emphysema, sleep apnea, bronchiectasis, previous tuberculosis, impaired diffusion capacity, hyperinflation and airway obstruction.

The common cut-of value < 0.7 for FEV_1_/FVC used for the definition of GOLD grades 1–4 versus COPD patients not fitting into these grades was used as an indicator of the presence of airway obstruction. In a similar manner, values ≥ 130%predicted of FRC/TLC were taken as indicator of lung hyperinflation, and values < 60%predicted of DLCO as indicator of impaired diffusion capacity; these values were obtained by receiver operating characteristic (ROC) analyses of mortality. In addition to these indicators, we included the clinical diagnoses of asthma, chronic bronchitis, lung emphysema, bronchiectasis, sleep apnoea and previous tuberculosis, all of them determined by structured interviews^15^. Other disorders such as interstitial lung disease, pulmonary embolism or pulmonary arterial hypertension were excluded from analysis due to their very low prevalence.

### Definition of the comorbidome

The comorbidome comprised the clinical diagnoses of arterial hypertension, myocardial infarction (MI), coronary artery disease (CAD) without MI, cardiac failure, gastroesophageal reflux disease (GERD), hyperlipidemia, gastric ulcers, liver cirrhosis, diabetes with insulin treatment, diabetes without insulin treatment, alcoholism, peripheral artery disease (PAD), osteoporosis, and mental disorders, determined by structured interviews. Other comorbidities were determined based on measured parameters, these being define kidney disease, hyperuricemia, cachexia, obesity, anemia or increased resting heart rate as a surrogate for sympathetic activity, cardiovascular risk factor and a predictor of all-cause mortality^22^. Kidney disease, was defined by an eGFR < 60 ml/min whereby the estimated glomerular filtration rate (eGFR) was calculated using the Chronic Kidney Disease Epidemiology Collaboration creatinine equation. Hyperuricemia was defined as urica acid ≥ 7 mg/dl, increased resting heart rate at a cutoff from ≥ 72 beats per minute (bmp)^22^, and anemia as hemoglobin (Hb) in women < 12 mg/dl and < 13 mg/dl in men, following the definition by the WHO^23^. Moreover cachexia was defined by body mass index (BMI) < 18.5 kg/m^2^ and obesity by BMI > 30 kg/m^2^, again following the World Health Organization (WHO) definition^24^.

### Charlson Comorbidity Index

The sum of the comorbidities was analyzed by the Charlson Comorbidity Index (CCI)^25^. Analogous to the analyses of the comorbidome and the pulmorbidome, renal disease was defined by an eGFR < 60 ml/min for which 1 score point was given; the definition of the other diseases was based on clinical diagnoses.

### Mortality

All-cause mortality was determined over a median follow-up period of 3.7 years (quartiles 1.8 and 4.5 years). If a patient missed a follow-up appointment without officially unsubscribing from the study, research assistants determined survival status by contacting partners, relatives, primary care physicians.

### Statistical analysis

Data in the tables are presented as numbers and percentages, or mean values and standard deviations (SD). Comparisons between men and women were performed with Student's t-test for numerical variables and chi-square statistics for categorical variables. The optimal cut-off values for predicting mortality were determined for DLCO and FRC/TLC using ROC curves and the corresponding Youden indices. Cox proportional hazard regression analysis was used to determine the prognostic value of the different variables of the comorbidome and pulmorbidome. Analyses were performed separately for men and women. In order to describe the predictive value of comorbidities per se, we did not include age in the prediction variables. Moreover, the analyses were repeated for patients of GOLD groups 1–4 only, to assess the impact of inclusion of patients at risk; the group of these patients was too small to perform a separate analysis.

Similar to the work by Divo et al.^10^ hazard ratios (HR) from the multivariate regression models were combined with the prevalence of each comorbidity. *p* values < 0.05 were considered as statistically significant. Statistical analyses were performed with SPSS version 25 (IBM Corp., Armonk, NY, USA). The comorbidome and pulmorbidome plots were created in Microsoft Excel.

### Ethics approval and consent to participate

The study was conducted in accordance with the amended Declaration of Helsinki. All assessments were approved by the central [Marburg (Ethikkommission FB Medizin Marburg)] and local [Bad Reichenhall (Ethikkommission bayerische Landesärztekammer); Berlin (Ethikkommission Ärztekammer Berlin); Bochum (Ethikkommission Medizinische Fakultät der RUB); Borstel (Ethikkommission Universität Lübeck); Coswig (Ethikkommission TU Dresden); Donaustauf (Ethikkommission Universitätsklinikum Regensburg); Essen (Ethikkommission Medizinische Fakultät Duisburg-Essen); Gießen (Ethikkommission Fachbereich Medizin); Greifswald (Ethikkommission Universitätsmedizin Greifswald); Großhansdorf (Ethikkommission Ärztekammer Schleswig–Holstein); Hamburg (Ethikkommission Ärztekammer Hamburg); MHH Hannover/Coppenbrügge (MHH Ethikkommission); Heidelberg Thorax/Uniklinik (Ethikkommission Universität Heidelberg); Homburg (Ethikkommission Saarbrücken); Immenhausen (Ethikkommission Landesärztekammer Hessen); Kiel (Ethikkommission Christian-Albrechts-Universität zu Kiel); Leipzig (Ethikkommission Universität Leipzig); Löwenstein (Ethikkommission Landesärztekammer Baden-Württemberg); Mainz (Ethikkommission Landesärztekammer Rheinland-Pfalz); München LMU/Gauting (Ethikkommission Klinikum Universität München); Nürnberg (Ethikkommission Friedrich-Alexander-Universität Erlangen Nürnberg); Rostock (Ethikkommission Universität Rostock); Berchtesgadener Land (Ethikkommission Land Salzburg); Schmallenberg (Ethikkommission Ärztekammer Westfalen-Lippe); Solingen (Ethikkommission Universität Witten-Herdecke); Ulm (Ethikkommission Universität Ulm); Würzburg (Ethikkommission Universität Würzburg] ethical committees and written informed consent was obtained from all patients.

## Results

### Baseline characteristics

Of 2741 patients enrolled in the COSYCONET cohort, 2575 patients (1531 men, 1044 women), were eligible for the current study. Men and women showed significant differences in age, BMI, smoking status, packyears (py), and several lung function parameters (FEV_1_%predicted, FEV_1_/FVC, FRC% %predicted, RV%predicted, FRC/TLC %predicted), but not DLCO. There were also significant differences in Hb and uric acid, but no in eGFR. A total of 400 patients did not meet the GOLD definition of grades 1–4, with women falling into this category more frequently than men and consistently having lower GOLD grades. There were, however, no differences between men and women in the GOLD groups Men had more comorbidities overall than women; the percentage of participants with a CCI > 2 was significantly higher in men, and this effect was independent of whether or not age was included in the calculation (see Table 1). Moreover, in men a CCI w/o age of > 2 versus ≤ 2 was linked to a mortality of 12.7% versus 9.5% (*p* = 0.062), while in women mortality in the two groups was 11.6% versus 4.1% (*p* < 0.001).

**Table 1:** Patient characteristics for the total study population and stratified for men and women.

	All N = 2575	Men N = 1531	Women N = 1044	*p* value
Age (years)	65.0 ± 8.6	65.8 ± 8.4	63.9 ± 8.7	< 0.001
BMI (kg/m^2^)	27.0 ± 5.3	27.5 ± 4.9	26.4 ± 5.8	< 0.001
Active Smokers	636 (24.7%)	349 (22.8%)	287 (27.5%)	0.006
Packyears	48.2 ± 36.0	53.3 ± 39.1	40.2 ± 28.6	< 0.001
FEV_1_ (%predicted)	56.8 ± 21.1	55.7 ± 21.0	58.4 ± 21.1	0.002
FEV_1_/FVC	55.3 ± 13.8	54.0 ± 13.7	57.2 ± 13.8	< 0.001
FRC (%predicted)	144.2 ± 37.5	140.9 ± 36.5	149.0 ± 38.6	< 0.001
RV (%predicted)	166.3 ± 53.2	162.9 ± 52.4	171.4 ± 54.0	< 0.001
FRC/TLC (%predicted)	115.8 ± 18.2	112.6 ± 17.9	120.5 ± 17.5	< 0.001
DLCO (%predicted)	58.8 ± 23.1	59.0 ± 22.7	58.6 ± 23.7	0.681
KCO (%predicted)	67.6 ± 23.7	67.7 ± 23.2	67.5 ± 24.6	0.882
Hemoglobin (g/dl)	14.6 ± 1.4	15.0 ± 1.4	14.1 ± 1.2	< 0.001
eGFR (ml/min)	82.2 ± 16.6	82.9 ± 16.9	82.6 ± 16.2	0.367
Uric acid (mg/dl)	5.95 ± 1.68	6.46 ± 1.59	5.18 ± 1.50	< 0.001
Heart frequency, beats per minute	72.8 ± 13.2	72.8 ± 13.5	72.9 ± 12.8	0.844
GOLD grade nd/1/2/3/4 (%)	15.5/7.6/35.5/32.2/9.2	12.8/7.7/36.3/33.2/10.1	19.7/7.5/34.2/30.7/8.0	< 0.001
GOLD group A/B/C/D (%)	40.0/24.2/13.5/21.9	41.3/24.6/13.1/20.4	38.1/23.5/13.9/24.0	0.210
CCI with age	4.00 ± 1.65	4.16 ± 1.67	3.76 ± 1.57	< 0.001
CCI > 2 w/o age	648 (25.2%)	433 (28.3%)	215 (20.6%)	< 0.001

Mean values and standard deviations are shown, or numbers and percentages. w/o = without, nd = not defined according to GOLD criteria. CCI = Charlson Comorbidity Index, computed either including age categories as conventional, or excluding age in order to focus on the number of comorbidities. *p* values refer to chi-square statistics from contingency tables or unpaired t-tests, dependent on the type of variable.

### Comorbidities

Among extra-pulmonary comorbidities, women showed significantly more often cachexia, mental disorders and osteoporosis. Men more often presented with arterial hypertension, coronary artery disease without infarction, myocardial infarction, hyperuricemia, diabetes with or without insulin therapy, alcoholism, peripheral artery disease, or anemia. No differences were found with respect to obesity, gastroesophageal reflux disease, gastric ulcer, hyperlipidemia, renal disease and increased heart rate. Among pulmonary comorbidities, women more often had asthma and severe hyperinflation, while men more often had sleep apnea, emphysema or airway obstruction. With regard to the diagnoses of chronic bronchitis, bronchiectasis, previous TB or impaired diffusion capacity, there were no sex-differences. Extra-pulmonary and pulmonary comorbidities are shown in Tables 2 and 3.

**Table 2:** Extra-pulmonary comorbidities.

	All N = 2575	Men N = 1531	Women N = 1044	*p*
Cachexia (BMI < 18.5 kg/m^2^)	78 (3.0%)	25 (1.6%)	53 (5.1%)	< 0.001
Obesity (BMI ≥ 30 kg/m^2^)	653 (25.4%)	403 (26.3%)	250 (23.9%)	0.174
Hypertension	1453 (56.4%)	899 (58.7%)	554 (53.1%)	0.004
Coronary artery disease w/o myocardial infarction	253 (9.8%)	193 (12.6%)	60 (5.7%)	< 0.001
Myocardial infarction	214 (8.3%)	171 (11.2%)	43 (4.1%)	< 0.001
Heart failure	139 (5.4%)	97 (6.3%)	42 (4.0%)	0.011
Gastro-esophageal reflux disease	383 (14.9%)	211 (13.8%)	172 (16.5%)	0.059
Hyperuricemia (UA ≥ 7 mg/dl)	622 (24.2%)	507 (33.1%)	115 (11.0%)	< 0.001
Gastric ulcer	312 (12.1%)	195 (12.7%)	117 (11.2%)	0.243
Liver cirrhosis	36 (1.4%)	23 (1.5%)	13 (1.2%)	0.585
Diabetes with insulin	137 (5.3%)	102 (6.7%)	35 (3.4%)	< 0.001
Diabetes w/o insulin	240 (9.3%)	173 (11.3%)	67 (6.4%)	< 0.001
Alcoholism	158 (6.1%)	126 (8.2%)	32 (3.1%)	< 0.001
Mental disorders	553 (21.5%)	247 (16.1%)	306 (29.3%)	< 0.001
Hyperlipidemia	1009 (39.2%)	614 (40.1%)	395 (37.8%)	0.247
Peripheral artery disease	298 (11.6%)	202 (13.2%)	96 (9.2%)	0.002
Osteoporosis	409 (15.9%)	146 (9.5%)	263 (25.2%)	< 0.001
Kidney disease (eGFR < 60)	264 (10.3%)	158 (10.3%)	106 (10.2%)	0.891
Anemia (13/12 mg/dl)	134 (5.2%)	97 (6.3%)	37 (3.5%)	0.002
Increased heart rate ≥ 72/min	1270 (49.3%)	753 (49.2%)	517 (49.5%)	0.866

Absolute numbers and percentages are given. *p* values refer to the comparison between women and men and were derived from Chi-square statistics.

**Table 3:** Pulmonary comorbidities, the “pulmorbidome”.

	All N = 2575	Men N = 1531	Women N = 1044	*p*
Asthma	4081 (18.7%)	228 (14.9%)	253 (24.2%)	< 0.001
Chronic bronchitis	1606 (62.4%)	950 (62.1%)	656 (62.8%)	0.687
Emphysema	275 (10.7%)	142 (9.3%)	133 (12.7%)	0.005
Sleep apnea	293 (11.4%)	219 (14.3%)	74 (7.1%)	< 0.001
Bronchiectasis	86 (3.3%)	55 (3.6%)	31 (3.0%)	0.388
Previous tuberculosis	58 (2.3%)	38 (2.5%)	20 (1.9%)	0.342
Impaired diffusion capacity *	1294 (54.6%)	779 (54.3%)	515 (55.1%)	0.697
Hyperinflation**	561 (22.4%)	250 (16.7%)	311 (30.7%)	< 0.001
Airway obstruction***	2175 (84.5%)	1336 (87.3%)	839 (80.4%)	< 0.001

Absolute numbers and percentages are given. *p* values refer to the comparison between women and men and were derived from Chi-square statistics. For 69 patients no TLC values were available, for 207 FRC/TLC values were missing. Impaired impaired diffusion capacity * was defined by DLCO ≤ 60, hyperinflation** by FRC/TLC ≥ 130%predicted and Airway obstruction*** by FEV_1_/FVC < 0.7.

### Associations of extra-pulmonary comorbidities (comorbidome) with mortality risk

**Figure 1:**
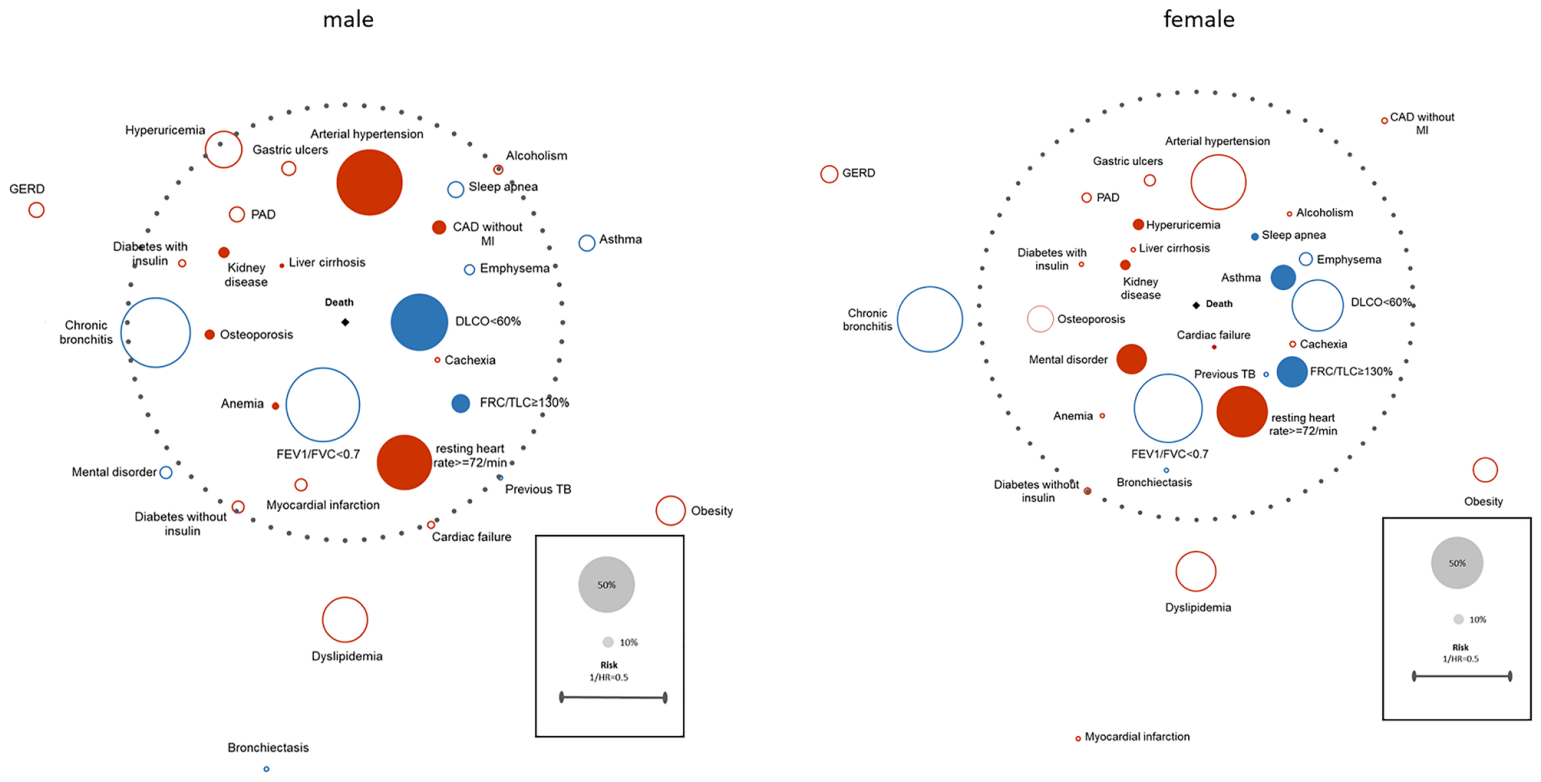
The left panel of this figure refers to men, the right panel to women. Each panel shows the hazard ratio as well as the prevalence of the comorbidities analyzed. Statistically significant associations (*p* < 0.05) are indicated by filled circles, those being not significant by open circles. Comorbidities that are part of the comorbidome are marked in red, those of the pulmorbidome in blue. The area of each circle represents the prevalence in the respective population. The dotted circle indicates a hazard ratio of 1. The distance of comorbidities from the circle and its center indicates the magnitude of the hazard ratio (see Supplemental Tables S1–S4), with hazard ratios greater than 1 plotted inwards and hazard ratios less than 1 outwards. This inverse type of plot was chosen to keep the figure compact.

During the median follow-up period of 3.7 years, 159 (10.4%) men and 59 (5.7%) women died. Using Cox regression analyses, only kidney disease (men HR 1.6; CI 1.0–2.4; women HR 2.6; CI 1.4–5.1; *p* < 0.05 each), and increased heart rate (men HR 1.4; CI 1.0–2.0; women HR 1.9; CI 1.1–3.2), were indicative for mortality risk in both, men and women. In men, arterial hypertension (HR 1.5; CI 1.1–2.2, *p* = 0.020), coronary artery disease without myocardial infarction (HR 1.6; CI 1.1–2.5, *p* = 0.028), liver cirrhosis (HR 2.6; CI 1.0–6.5, *p* = 0.047) and osteoporosis (HR 1.6; CI 1.0–2.5, *p* = 0.041) were associated with increased mortality risk. In women, heart failure (HR 4.7; CI 2.0–11.1, *p* < 0.001), mental disorders (HR 2.6; CI 1.5–4.5, *p* < 0.001) and hyperuricemia (HR 2.2; CI 1.1–4.3, *p* = 0.029) turned out to be independent risk factors for mortality. Figure 1 shows the results for mortality risk of different extra-pulmonary comorbidities for men and women in terms of HR (red colour). The numerical results for men and women are shown in the Additional files S1 and S2.

### Associations of pulmonary comorbidities (pulmorbidome) with mortality risk

Cox proportional regression analysis of pulmonary diseases showed that severe hyperinflation was a risk factor for mortality in both sexes (men HR 1.5; CI 1.0–2.3; women HR1.9; CI 1.0–3.4; *p* < 0.05 each). In addition, impaired DLCO was a relevant risk factor only in men (HR 2.9; CI 1.9–4.5; *p* < 0.001). Moreover, in women the presence of asthma (HR 2.4; CI 1.3–4.3; *p* = 0.004) or sleep apnea (HR2.4; CI 1.0–5.7; *p* = 0.05) were relevant risk factors, whereas men did not show an increased risk associated with these diseases. Figure 1 shows the results for mortality risk separately for men and women in terms of HR (blue colour). We repeated these analyses for GOLD 1–4 patients only, and no significant changes in HR were observed. The numerical results for all patients or the GOLD 1–4 sub-group are shown in tables S3 and S4 in the Additional files.

## Discussion

We analyzed sex-specific associations of COPD comorbidities with mortality, categorizing comorbidities into comorbidome and pulmorbidome. In line with known data, the frequency of specific disorders showed significant differences between sexes. The major result, however, was that such differences were also evident in the association of comorbidities with mortality. These differences were not explained by mere differences in prevalence.

Gender-differences in COPD are well known for a number of prognostically relevant factors, such as symptoms and exacerbations^5,8^. Unfortunately, however, women often comprised a small proportion of COPD studies. For example, the COTE cohort and the UPLIFT, WISDOM, FLAME studies included only 11, 25, 17, 23% women, respectively^10,26–28^. In contrast, we had 40.5% women in our cohort and thus could perform separate analyses. In real life, patients are often diagnosed and treated as COPD patients^16^ even if not fulfilling the criteria of GOLD grades 1–4^29^. We thus included a large number of patients diagnosed with COPD but not categorized into in GOLD 1–4^16^. Noteworthy, the results did not depend on their inclusion, which suggests that our findings apply to broad populations of patients diagnosed with COPD in clinical practice.

Some of the respiratory disorders that were part of the pulmorbidome showed differences between men and women in their association with mortality, as illustrated in Fig. 1. In line with the literature, lung hyperinflation was a risk factor for both, men and women^30^. In contrast, impaired DLCO, occurring in 54% of men and 55% of women, was associated with mortality only in men, while asthma, diagnosed in 14.9% of men and 24.2% of women, was associated with mortality only in women.

The differences in the frequencies of comorbidities probably result from a number of factors, such as the diagnostic scope and expectations of treating physicians^31^, including a different role for lung cancer screening by computed tomography^32^. Moreover, anatomical conditions including smaller lung and airways in women may play a role, as well as the effect of sex hormones on airway hyperresponsiveness^33^. The differences also include a potentially different impact of risk factors, especially smoking. Increased airway hyperresponsiveness as observed in women leads to higher susceptibility to cigarette smoke, resulting in stronger lung function decline compared to men^34^. Inline with other COPD cohorts^12,35^, we found women to be more often diagnosed with concomitant asthma than men, and this was associated with an increased mortality risk in women but not in men. When considering the fact that for the asthma-COPD overlap syndrome the results of previous studies are not fully consistent^36,37^, our findings raise the possibility that this could be due to different proportions of women and men in the different studies.

When examining the decline of DLCO in smokers with and without COPD, Casanova and colleagues found lower baseline values and a more rapid decline in women, indicating a role for gender in the course of gas exchange in COPD^38^. In contrast, our population showed no differences in average DLCO or the prevalence of reduced DLCO values between men and women. Despite this, impaired DLCO was associated with mortality only in men, in line with the results of a study in which most participants were men^39^. As cardiac function may be affected by oxygen supply and cardiac disease was more frequent in men, it might be hypothesized that low DLCO had an effect via reduced tissue oxygenation.

Following the work of Divo and colleagues, we summarized a number of extra-pulmonary comorbidities into a comorbidome^10^, however including only comorbidities that were not specific for sex. This allowed for a comparison between men and women that relied on generic and COPD-related comorbidities only. In men, arterial hypertension and coronary heart disease as well as liver cirrhosis and osteoporosis were linked to mortality, in women heart failure and mental illness. This demonstrated that also comorbidities not specific for gender showed a different role in prognosis.

It is of interest to compare the frequencies of comorbidities in our cohort with those of other cohorts. In the ECCO study, women had a higher prevalence of heart failure^3^, but in our cohort the prevalence was slightly lower (4.0 vs. 6.3%). Irrespective of this, the negative effect on survival occurred only in women. A possible explanation could be the difference in the causes of heart failure between men and women; in most men, a primary ischemic origin can be assumed, which suggests a large overlap between the diagnoses of heart failure and coronary artery disease. As known from cardiac cohorts, heart failure in women in the relevant age group is more often non-ischemic compared to men^40^. The factors underlying the association with heart failure in women are unknown but women with heart failure often report a higher symptom burden than men, are more likely to suffer from depression, and tolerate pharmacologic heart failure therapies less well than men^41^.

Mental illness, especially depression, was diagnosed significantly more frequently in women than in men and was also predictive for mortality exclusively in women. The association between mortality and depression may be linked to insufficient healthcare utilization and poorer treatment compliance^42^. On the other hand, it should be noted that depressive symptoms reflected in diagnostic scores can interfere with COPD symptoms and comorbidities. A previous analysis of COSYCONET data showed that this interference could partially explain high depression scores in COPD^43^.

Interestingly, hyperuricemia was associated with higher mortality in the total study population, specifically in women but not in men. The majority of investigations did not examine men and women separately^44,45^. This result is in line with previous studies showing an association between hyperuricemia and diastolic dysfunction and major cardiovascular events only in women^46,47^. The exact causes of this difference are unclear, but sex hormones, especially estrogene as uricosuric agent, could play a role together with the effects of tobacco smoking^48^.

For osteoporosis we observed an association with mortality only in men, although this comorbiditiy was more common in women. One possible reason for this difference could be that in men osteoporosis is diagnosed at later and more advanced stages, even in COPD patients known to be at increased risk for osteoporosis. Similarly, for liver cirrhosis a negative effect on survival was found only in men, although it was diagnosed with equal frequency in men and women. One possible reason could be that women with cirrhosis tend to have fewer complications of this disease and lower rates of hepatocellular carcinoma^49^.

Anemia is known as risk factor for mortality in COPD. For diagnosis, we used sex-specific hemoglobin cut-off values. According to these criteria, anemia occurred in 6.3% of men and 3.5% of women (Table 2). Again, a negative effect on survival could only be demonstrated for men. One explanation for this finding cut be inadequacy of the cut-off values, another a gender-specific interaction of anemia with concomitant diseases. Data from the SPIROMICS cohort showed that anemia in COPD was associated with poorer exercise capacity, greater dyspnea and higher disease severity and that these effects were particularly evident in individuals with chronic cardiac and metabolic diseases as comorbidities^50^. It is also plausible to assume that anemia affects patients with ischemic heart disease more than those without, suggesting that the importance of common risk factors for COPD mortality may be modulated by comorbidities that show different prevalence or type in men and women.

## Limitations

Although the cross-sectional design enabled us to determine the correlations between comorbidities and mortality, direct causal relationships cannot be inferred. Although mortality was not high within the follow-up period, the size of the cohort and the balanced gender distribution allowed for a detailed assessment of gender differences. A further limitation of our study is the lack of information on the cause of death, therefore mortality was taken as all-cause mortality. According to the study protocol of COSYCONET the presence of malignant disease was an exclusion criterion, thus we could not consider these diseases in our risk assessment. Another limitation was that comorbidities could not be validated by independent assessments and were based on physician-based diagnoses within a structured interview, a gender bias such as systematic under- or overdiagnosis in one of the two sexes is possible.

## Conclusions

Using the same type of analysis in men and women with COPD, we found marked sex differences in the associations of comorbidities with mortality. Regarding the pulmorbidome, which representing respiratory disorders, the differences referred to asthma, sleep apnoea, diffusing capacity and lung hyperinflation. Regarding the comorbidome representing non-respiratory disorders, arterial hypertension, coronary artery disease, hyperuricemia, anemia, osteoporosis, mental disorders and liver cirrhosis played a different role in men and women. Increased HR and kidney disease were risk factors in both groups. These data demonstrate that not only the prevalence of comorbidities but also their impact on mortality differs between sexes. It has to be explored to which extent this can lead to more individually tailored strategies for the treatment and surveillance of COPD patients.

## Supplementary Information

Supplementary Tables.

## Data Availability

Data may be obtained from a third party and are not publicly available. The full dataset supporting the conclusions of this article is available upon request and application from the Competence Network Asthma and COPD (ASCONET, http://www.asconet.net/html/cosyconet/projects).

## Abbreviations

BMI: Body mass index
CAD: Coronary artery disease
CCI: Charlson Comorbidity Index
CKD: Chronic kidney disease
COPD: Chronic obstructive pulmonary disease
COSYCONET: COPD and SYstemic consequences-COmorbidities NETwork
DLCO: Diffusion capacity of the lungs for carbon monoxide
ECSC: European Coal and Steel Community
eGFR: Estimated glomerular filtration rate
FEV_1_: Forced expiratory volume in 1 s
FRC: Functional residual capacity
FVC: Forced vital capacity
GLI: Global Lung Function Initiative
GOLD: Global Initiative for Chronic Obstructive Lung Disease
Hb: Hemoglobin
HR: Hazard ratios
MI: Myocardial infarction
mMRC: Medical Research Council
py: Pack years
ROC: Receiver operating characteristic
TLC: Total lung capacity
WHO: World Health Organization
