# Supplementary material for: Sex-specific associations of comorbidome and pulmorbidome with mortality in chronic obstructive pulmonary disease: results from COSYCONET
Source: Sci Rep. 2022 May 24;12:8790. doi: 10.1038/s41598-022-12828-8 (PMC9130231; doi:10.1038/s41598-022-12828-8)
Supplement: Supplementary file 1 — Supplementary Tables. [file 41598_2022_12828_MOESM1_ESM.docx]

|  |  |  |  |  | **95%CI of HR** | |
| --- | --- | --- | --- | --- | --- | --- |
| **Predictor** | **B** | **SE** | **p-value** | **HR** | **lower** | **upper** |
| Cachexia (BMI < 18.5 kg/m^2^) | 0.779 | 0.522 | 0.136 | 2.179 | 0.783 | 6.063 |
| Obesity (BMI ≥ 30 kg/m^2^) | -0.547 | 0.212 | 0.010 | **0.579** | 0.382 | 0.876 |
| Arterial hypertension | 0.428 | 0.184 | 0.020 | **1.534** | 1.069 | 2.201 |
| Coronary artery disease without remote infarction | 0.487 | 0.222 | 0.028 | **1.628** | 1.054 | 2.514 |
| Myocardial infarction | 0.259 | 0.266 | 0.330 | 1.296 | 0.769 | 2.183 |
| Left heart failure | -0.006 | 0.307 | 0.984 | 0.994 | 0.544 | 1.814 |
| Gastroesophageal reflux disease | -0.411 | 0.263 | 0.118 | 0.663 | 0.396 | 1.110 |
| Hyperuricemia (uric acid ≥ 7 mg/dL) | 0.031 | 0.174 | 0.859 | 1.031 | 0.733 | 1.451 |
| Gastric ulcer | 0.286 | 0.211 | 0.176 | 1.331 | 0.880 | 2.014 |
| Liver cirrhosis | 0.940 | 0.474 | 0.047 | **2.561** | 1.011 | 6.487 |
| Diabetes with insulin therapy | 0.226 | 0.286 | 0.430 | 1.254 | 0.715 | 2.198 |
| Diabetes without insulin therapy | 0.020 | 0.261 | 0.938 | 1.021 | 0.612 | 1.703 |
| Alcohol addiction | 0.007 | 0.285 | 0.982 | 1.007 | 0.575 | 1.761 |
| Mental disorders including depression | -0.071 | 0.231 | 0.760 | 0.932 | 0.592 | 1.466 |
| Hyperlipidemia | -0.311 | 0.178 | 0.081 | 0.733 | 0.517 | 1.039 |
| Circulatory disorders legs | 0.355 | 0.208 | 0.088 | 1.426 | 0.949 | 2.144 |
| Osteoporosis | 0.471 | 0.230 | 0.041 | **1.601** | 1.020 | 2.514 |
| Chronic kidney disease (eGFR < 60 mL/min) | 0.442 | 0.224 | 0.048 | **1.556** | 1.004 | 2.411 |
| Anemia (hemoglobin < 13 g/dL in men) | 0.696 | 0.249 | 0.005 | **2.006** | 1.231 | 3.269 |
| Sympathic activity (resting heart frequency ≥ 72/min) | 0.362 | 0.164 | 0.027 | **1.436** | 1.042 | 1.979 |

**Table S1:** Comorbidome in men in relation to mortality according to the results of Cox proportional hazard regression analysis. B = regression coefficient, SE = its standard error, HR = hazard ratio (=exp(B)), 95%CI = its 95% confidence interval

|  |  |  |  |  | **95%CI of HR** | |
| --- | --- | --- | --- | --- | --- | --- |
| **Predictor** | **B** | **SE** | **p-value** | **HR** | **lower** | **upper** |
| Cachexia (BMI < 18.5 kg/m^2^) | 0.736 | 0.542 | 0.175 | 2.087 | 0.721 | 6.035 |
| Obesity (BMI ≥ 30 kg/m^2^) | -0.429 | 0.339 | 0.206 | 0.651 | 0.335 | 1.267 |
| Arterial hypertension | 0.534 | 0.298 | 0.073 | 1.706 | 0.951 | 3.059 |
| Coronary artery disease without remote infarction | -0.202 | 0.511 | 0.692 | 0.817 | 0.300 | 2.223 |
| Myocardial infarction | -0.740 | 0.752 | 0.325 | 0.477 | 0.109 | 2.085 |
| Left heart failure | 1.557 | 0.432 | 0.000 | **4.745** | 2.033 | 11.074 |
| Gastroesophageal reflux disease | -0.585 | 0.412 | 0.156 | 0.557 | 0.249 | 1.249 |
| Hyperuricemia (uric acid ≥ 7 mg/dL) | 0.772 | 0.354 | 0.029 | **2.164** | 1.081 | 4.333 |
| Gastric ulcer | 0.476 | 0.361 | 0.187 | 1.610 | 0.794 | 3.264 |
| Liver cirrhosis | 0.941 | 0.671 | 0.161 | 2.561 | 0.688 | 9.543 |
| Diabetes with insulin therapy | 0.577 | 0.581 | 0.321 | 1.780 | 0.570 | 5.559 |
| Diabetes without insulin therapy | 0.000 | 0.636 | 1.000 | 1.000 | 0.287 | 3.481 |
| Alcohol addiction | 0.498 | 0.623 | 0.424 | 1.646 | 0.485 | 5.586 |
| Mental disorders including depression | 0.945 | 0.285 | 0.001 | **2.573** | 1.473 | 4.495 |
| Hyperlipidemia | -0.217 | 0.292 | 0.457 | 0.805 | 0.454 | 1.426 |
| Circulatory disorders legs | 0.336 | 0.372 | 0.367 | 1.399 | 0.675 | 2.902 |
| Osteoporosis | 0.331 | 0.287 | 0.249 | 1.392 | 0.793 | 2.443 |
| Chronic kidney disease (eGFR < 60 mL/min) | 0.971 | 0.340 | **0.004** | 2.640 | 1.355 | 5.145 |
| Anemia (hemoglobin <12 g/dL in women) | 0.397 | 0.564 | 0.482 | 1.487 | 0.492 | 4.490 |
| Sympathic activity (resting heart frequency ≥ 72/min) | 0.619 | 0.285 | **0.030** | 1.856 | 1.062 | 3.247 |

**Table S2:** Comorbidome in women in relation to mortality according to the results of Cox proportional hazard regression analysis. B = regression coefficient, SE = its standard error, HR = hazard ratio (=exp(B)), 95%CI = its 95% confidence interval

|  |  |  |  |  | **95%CI of HR** | |
| --- | --- | --- | --- | --- | --- | --- |
| **Predictor** | **B** | **SE** | **p-value** | **HR** | **lower** | **upper** |
| Asthma | -0.158 | 0.260 | 0.544 | 0.854 | 0.513 | 1.423 |
| Chronic bronchitis | 0.136 | 0.182 | 0.454 | 1.146 | 0.802 | 1.638 |
| Emphysema | 0.477 | 0.252 | 0.058 | 1.612 | 0.984 | 2.640 |
| Bronchiectasis | -0.734 | 0.585 | 0.210 | 0.480 | 0.153 | 1.511 |
| Sleep apnea | 0.229 | 0.240 | 0.340 | 1.257 | 0.785 | 2.013 |
| History of tuberculosis | -0.008 | 0.466 | 0.986 | 0.992 | 0.398 | 2.472 |
| Gas exchange impairment (DLCO < 60 %predicted GLI) | 1.071 | 0.218 | 0.000 | **2.919** | 1.904 | 4.477 |
| Lung hyperinflation (ITGV/TLC ≥ 130 %predicted ECSC) | 0.433 | 0.202 | 0.032 | **1.542** | 1.039 | 2.290 |
| Airway obstruction (FEV_1_/FVC < 0.7) | 0.938 | 0.516 | 0.069 | 2.554 | 0.929 | 7.021 |

**Table S3:** Pulmorbidome in men in relation to mortality according to the results of Cox proportional hazard regression analysis. B = regression coefficient, SE = its standard error, HR = hazard ratio (=exp(B)), 95%CI = its 95% confidence interval, DLCO = diffusing capacity for carbon monoxide, ITGV = intrathoracic gas volume, TLC = total lung capacity, FEV_1_ = forced expiratory volume in 1 second, FCV = forced vital capacity

|  |  |  |  |  | **95%CI of HR** | |
| --- | --- | --- | --- | --- | --- | --- |
| **Predictor** | **B** | **SE** | **p-value** | **HR** | **lower** | **upper** |
| Asthma | 0.864 | 0.302 | **0.004** | 2.373 | 1.313 | 4.291 |
| Chronic bronchitis | -0.204 | 0.299 | 0.495 | 0.815 | 0.454 | 1.465 |
| Emphysema | 0.596 | 0.343 | 0.082 | 1.815 | 0.927 | 3.553 |
| Bronchiectasis | 0.245 | 0.730 | 0.737 | 1.278 | 0.306 | 5.343 |
| Sleep apnea | 0.869 | 0.444 | 0.050 | 2.383 | 0.998 | 5.690 |
| History of tuberculosis | 0.786 | 0.739 | 0.287 | 2.194 | 0.516 | 9.331 |
| Gas exchange impairment (DLCO < 60 %predicted GLI) | 0.581 | 0.344 | 0.091 | 1.787 | 0.911 | 3.505 |
| Lung hyperinflation (ITGV/TLC ≥ 130 %predicted ECSC) | 0.617 | 0.314 | **0.049** | 1.854 | 1.003 | 3.427 |
| Airway obstruction (FEV_1_/FVC < 0.7) | 0.697 | 0.627 | 0.266 | 2.008 | 0.588 | 6.855 |

**Table S4:** Pulmorbidome in women in relation to mortality according to the results of Cox proportional hazard regression analysis. B = regression coefficient, SE = its standard error, HR = hazard ratio (=exp(B)), 95%CI = its 95% confidence interval, DLCO = diffusing capacity for carbon monoxide, ITGV = intrathoracic gas volume, TLC = total lung capacity, FEV_1_ = forced expiratory volume in 1 second, FCV = forced vital capacity
